# Shoulder pain in amateur fast bowlers: A case example illustrating opportunities to embed prevention in undergraduate physiotherapy education

**DOI:** 10.4102/sajp.v82i2.2304

**Published:** 2026-04-30

**Authors:** Muhammad A. Dawood, Benita Olivier, Maria E. Cochrane

**Affiliations:** 1 Department of Physiotherapy, School of Health Care Sciences, Sefako Makgatho Health Sciences University, Ga-Rankuwa, South Africa; 2 Wits Cricket Research Hub for Science, Medicine and Rehabilitation, Faculty of Health Sciences, University of the Witwatersrand, Johannesburg, South Africa; 3 Department of Physiotherapy, Faculty of Health Sciences, University of the Witwatersrand, Johannesburg, South Africa; 4 Centre for Healthy Living Research, Oxford Institute of Applied Health Research, Faculty of Health, Science and Technology, Oxford Brookes University, Oxford, United Kingdom; 5 Department of Health Professions Education, Faculty of Health Sciences, University of Pretoria, Pretoria, South Africa

**Keywords:** physiotherapy education, prevention, injury risk factors, shoulder pain, cricket, Sustainable Development Goal 3, undergraduate curriculum, sports physiotherapy

## Abstract

**Background:**

Health professions education is undergoing a paradigm shift from a predominantly curative focus towards prevention and health promotion, in line with global health priorities such as Sustainable Development Goal 3 (SDG3). Physiotherapy curricula, however, often still devote limited attention to preventative competencies.

**Objectives:**

The objectives of this study are to identify risk factors for shoulder pain in amateur fast bowlers and illustrate how such evidence can inform the integration of preventative competencies into undergraduate physiotherapy curricula.

**Method:**

A prospective, longitudinal cohort study was conducted during the 2018–2019 amateur cricket season. Thirty-nine male fast bowlers completed baseline musculoskeletal screening and weekly online questionnaires documenting injury incidence. Physical assessments included shoulder range of motion, scapular control, muscle strength, endurance and stability tests. Data were analysed using *t*-tests, Mann–Whitney U tests, chi-square and/or Fisher’s exact tests.

**Results:**

Nine participants (23%) sustained shoulder pain during the season. Risk factors associated with injury included scapular dyskinesia, decreased internal rotation, increased external rotation, glenohumeral internal rotation deficit, poor endurance and reduced isometric muscle strength. These factors demonstrated moderate-to-large effect sizes.

**Conclusion:**

The identification of modifiable risk factors for shoulder pain demonstrates the value of embedding screening skills into undergraduate physiotherapy curricula. Teaching students to assess and address risk factors fosters a preventative orientation that extends beyond sports injuries to broader health contexts.

**Clinical implications:**

Incorporating risk factor screening into training equips physiotherapy graduates with practical skills for early detection and prevention, thereby reducing the burden of preventable conditions and aligning education with the objectives of SDG3.

## Introduction

Health professions education is experiencing a paradigm shift away from curative models towards more proactive, preventative models (Babatunde et al. 2021). Although curative healthcare still has its place in undergraduate health sciences curricula, a sole focus on curative health results in high cases of preventable diseases, injuries and mortalities (Babatunde et al. 2021). Preventative models, in contrast, contribute to improved population health and well-being, aligning with the Sustainable Development Goals (SDGs), particularly Sustainable Development Goal 3 (SDG3) (Lakioti et al. 2025).

Physiotherapy education has not remained static in the face of evolving healthcare needs.

Curricula worldwide have begun to emphasise preventative competencies, preparing graduates who are equally adept at injury risk reduction and long-term health promotion as they are at rehabilitation (Beiling & Chisati 2017; Janse van Vuuren 2022). This transformation reflects broader trends in health professions education, where responsiveness to societal needs, population health priorities and sustainable healthcare delivery are recognised as markers of quality and relevance (Thibault 2020). The need to transform physiotherapy education in South Africa, from curative to preventative, was identified as early as 2002 (Krause 2002). Although substantial curriculum reform has taken place since the early 2000s, the need to broaden the role of the physiotherapist in primary healthcare remains (Narain & Mathye 2023). Currently, the Health Professions Council of South Africa’s (HPCSA) minimum training standards for physiotherapists include preventative health and health promotion as training requirements for undergraduate students (HPCSA n.d.).

However, this component of undergraduate training comprises less than 4% of the recommended curriculum and is not specific to particular subject areas in physiotherapy practice.

Our case study of shoulder pain among amateur fast bowlers exemplifies the practical value of embedding prevention into different subject areas within physiotherapy curricula. The risk factors identified are not only clinically relevant but also provide concrete learning opportunities for students. The case study serves as an example and can, and should, be extrapolated to other subject and clinical areas within physiotherapy practice.

By teaching future physiotherapists to recognise risks, education moves beyond preparing graduates to manage conditions, equipping them instead to prevent injuries and conditions in community populations.

This study did not evaluate an educational intervention. Instead, we present the findings as a case example to demonstrate how clinical evidence can inform preventative training within undergraduate physiotherapy curricula.

## Research methods and design

The methodology used to determine the incidence of, and factors associated with, shoulder pain in amateur fast bowlers is discussed in this section.

A prospective, longitudinal cohort study design was employed (Dallinga et al. 2019). At the time of the study, 12 amateur cricket teams were affiliated with the Northern Cricket Union Premier Cricket League, and these were targeted for player recruitment. To ensure uniformity during data collection, all assessments were conducted at the clubhouses of the respective cricket clubs. Amateur cricket players were recruited on a voluntary basis. Players were considered for inclusion in the study if they were fast bowlers, 18 years or older, free of pain and/or injury and actively participated in the 2018–2019 amateur cricket season. Players were excluded from participation if they had undergone any surgery to the shoulder or presented with progressive musculoskeletal disease. Ultimately, 39 players were enrolled.

At baseline (pre-season), participants completed an online questionnaire through the REDCap system (Harris et al. 2009) and underwent standardised musculoskeletal screening. The questionnaire collected demographic and anthropometric data, while physical screening provided baseline musculoskeletal measurements. All screening tests were conducted within a three-week period prior to the start of the 2018–2019 cricket season.

All physical screening procedures were performed by the principal investigator, a qualified physiotherapist with experience in musculoskeletal assessment. The physical screening consisted of:

- Shoulder internal rotation (IR) and external rotation (ER) range of motion measurements (ROM), with the shoulder in 90° abduction (Kolber & Hanney 2012).
- Weighted and non-weighted scapula dyskinesia testing (SDT) (McClure, Greenberg & Kareha 2012).
- Isometric strength testing with a handheld dynamometer (specifically for shoulder ER and IR at 90° abduction).
- Protraction of the scapula with the arm at 90° forward flexion (in supine).
- Abduction in sitting at 45°.
- Fatigue testing in two positions (scaption and prone-Y) (Blazquez et al. 2013; Clarsen et al. 2014).
- Closed kinetic chain upper extremity stability test (CKUEST) (Tucci et al. 2014).

Throughout the season, participants completed weekly online questionnaires reporting injuries sustained, body region affected and type of injury.

From the data collected with the questionnaires and physical screening, injury incidence was calculated for the 2018–2019 season. Only non-contact shoulder pain or injuries were included in the statistical analysis, as these aligned with the risk factors under investigation. For the purpose of this study, shoulder injury was defined as ‘any shoulder pain (time-loss and/or non-time-loss) that required medical attention and had the potential to affect cricket training or playing’ (Orchard, Kountouris & Sims 2016).

Data analysis was performed using IBM^®^ Statistical Package for Social Sciences (SPSS) statistics (version 26). Independent *t*-tests were used to analyse the differences between the two groups in the normally distributed continuous variables. Mann–Whitney U tests were used to analyse the continuous variables that were not normally distributed. Categorical variables were analysed for associations by means of either a chi-square test or a Fischer’s exact test (Nachar 2008).

No missing data were encountered.

The following effect size calculations were applied: Hedge’s G (for the parametric continuous variables), probability of superiority (for the non-parametric continuous variables) and Phi coefficient (for the non-parametric categorical variables) (Dutton, Tam & Gray 2019). An effect size of 0.1 is judged to have a small effect, 0.3 a medium effect and 0.5 a large effect on outcomes.

An effect size of 0.4 is the hinge point, an effect size at which an initiative can be said to be having a ‘greater than average influence’ on the outcome (in this case, shoulder pain).

### Ethical considerations

Ethical clearance to conduct this study was obtained from the University of the Witwatersrand Research and Ethics Committee (No. M180410). Prior to conduction of the study, permission to conduct the study was granted by the Northern Cricket Union Premier Cricket League and the National Cricket Board. All participants provided written, voluntary informed consent. The research was conducted in accordance with the Helsinki Declaration.

## Results

Forty-three male fast bowlers (all-rounders included) completed the baseline screening procedures. Four were excluded (left the study [*n* = 3], stopped playing cricket [*n* = 1]). This resulted in a sample of 39 amateur fast bowlers (mean age 23.9 years, mean body mass index 25.3 kg/m^2^, pace bowlers *n* = 13, all-rounders *n* = 26, right dominant *n* = 37 and left dominant *n* = 2).

There were nine incidents of shoulder pain (23% of the population) among the 39 amateur fast bowlers during the 2018–2019 season. Of the nine participants who reported injuries, three sustained time-loss injuries. The injuries that were reported occurred by insidious onset (2.2%), gym activities (1.1%) and during practice and match play (96.7%).

Factors associated with the development of shoulder pain were determined based on analysis of data from the physical screening. Table 1, Table 2 and Table 3 indicate that scapula dyskinesia, decreased IR ROM, increased Glenohumeral Internal Rotation Deficit (GIRD), increased ER and poor endurance and isometric muscle strength of selected muscles surrounding the scapula (i.e. deltoid, serratus anterior and the rotator cuff) displayed an effect on the development of shoulder pain (moderate-to-large effect sizes of 0.3 and higher). The weight and height of the fast bowlers showed no influence on the development of shoulder pain.

**TABLE 1:** A comparison of the parametric continuous variables between the fast bowlers with shoulder pain and the fast bowlers without shoulder pain (*n* = 39).

Variable	Without shoulder pain (*n* = 30)		With shoulder pain (*n* = 9)		*p*-value	Effect size (Hedge’s G)
	Mean	s.d.	Mean	s.d.		
Weight	83.9	13.60	83.1	14.0	0.90	0.10
Height	1.8	0.74	1.8	0.1	0.60	0.00
CKUEST NS	10.6	2.00	10.0	1.9	0.44	0.30
S RC R	15.4	4.40	16.3	3.3	0.58	0.20
S SA L	24.0	4.30	23.8	4.0	0.90	0.10
PASS ER	87.6	13.50	101.4	12.5	0.01	1.00
PASS IR	56.1	17.20	46.8	13.9	0.15	0.60
ACT ER	78.0	14.30	93.7	14.0	0.00	1.08
ACT IR	46.0	15.30	39.1	12.2	0.23	0.50
BS scaption position	11.0	6.20	12.6	5.7	0.51	0.30

CKUEST, closed kinetic upper extremity test; S, isometric strength test; RC, rotator cuff; R, right; L, left; MR, medial rotation; D, deltoid; SA, serratus anterior; PASS, passive ROM; ER, external rotation; IR, internal rotation; ACT, active ROM; BS, Borg scale; ROM, range of motion measurements; s.d., standard deviation.

**TABLE 2:** A comparison of the non-parametric continuous variables between the fast bowlers with shoulder pain and the fast bowlers without shoulder pain (*n* = 39).

Variable	Without shoulder pain (*n* = 30)		With shoulder pain (*n* = 9)		*p*-value	Effect size (probability of superiority)
	Median	IQR	Median	IQR		
CKUEST NO	18.00	6.0	18.0	3.9	0.67	0.5
CKUEST PO	69.70	27.0	67.1	24.6	0.63	0.5
S RC L	13.25	5.3	14.0	2.5	0.68	0.5
S MR L	13.00	3.9	10.6	4.8	0.59	0.5
S MR R	12.60	6.3	11.4	2.4	0.11	0.3
S D L	19.35	5.5	21.7	6.7	0.70	0.5
S D R	19.30	6.0	20.0	6.1	0.68	0.5
S SA R	24.30	6.0	24.1	4.5	0.75	0.5
ARC PASS	150.50	21.0	143.0	22.0	0.46	0.4
ARC ACT	129.50	27.0	132.0	17.0	0.80	0.5
BS prone-Y position	5.00	4.0	6.0	5.0	0.55	0.4

IQR, interquartile range; CKUEST, closed kinetic upper extremity test; NO, number of touches; PO, power score; S, isometric strength test; RC, rotator cuff; R, right; L, left; MR, medial rotation; D, deltoid; SA, serratus anterior; PASS, passive ROM; ER, external rotation; IR, internal rotation; ACT, active ROM; BS, Borg scale; ROM, range of motion measurements.

**TABLE 3:** A comparison of the categorical variables between the fast bowlers with shoulder pain and those without shoulder pain (*N* = 39).

Variable	Outcome	Without shoulder pain (*n* = 30)		With shoulder pain (*n* = 9)		*p*-value	Effect size (Phi-coefficient)
		*n*	%	*n*	%		
SH injury history	No	13	43	4	44	1.00	0.20
	Yes	17	57	5	56	1.00	-
SDT W flexion	Abnormal	22	73	7	78	1.00	0.20
	Normal	8	27	2	22	1.00	-
SDT W abduction	Abnormal	12	40	4	44	0.59	0.04
	Normal	18	60	5	56	0.59	-
SDT flexion	Abnormal	14	47	7	78	0.01	0.30
	Normal	16	63	2	22	0.01	-
SDT abduction	Abnormal	12	40	5	56	0.47	0.10
	Normal	18	60	4	44	0.47	-

**SDT total**	**Abnormal**	**19**	**63**	**7**	**78**	**0.69**	**0.10**
	**Normal**	**11**	**27**	**2**	**22**	**0.69**	-

SDT, scapula dyskinesia test; W, weighted; SH, shoulder.

## Discussion

Although the study itself was a clinical investigation, its findings illustrate the types of risk factor information that could be used to strengthen prevention-focused teaching in undergraduate programmes. This study contributes to the growing body of evidence emphasising the need for a paradigm shift in physiotherapy education, from predominantly curative training towards prevention-focused curricula (World Physiotherapy 2022).

Traditionally, undergraduate physiotherapy programmes have prioritised rehabilitation of established impairments and injuries, with comparatively little emphasis on risk factor identification and early intervention (Shumba & Tekian 2024). While rehabilitation remains an essential professional role, the findings from this study illustrate that many shoulder injuries in amateur fast bowlers were preceded by identifiable, modifiable risk factors. The physical assessment techniques that were used on the participants in the study are taught to undergraduate physiotherapy students as part of the standard skill set they are expected to acquire during the course of their degree. However, currently, these tests are taught as part of the battery of tests that need to be performed on a patient who has already sustained an injury and is in the recovery phase of healing.

Teaching students to recognise and address risk factors, using skills they are familiar with, offers a pathway to preventing injuries before they occur, thereby reducing both personal and health system burden (Zosel et al. 2021).

Our case study demonstrates that factors such as reduced IR, scapular dyskinesia, poor endurance and altered muscle strength are associated with the development of shoulder pain. Each of these risk factors can be assessed with relatively simple, low-cost screening tools that are either already taught to undergraduate students or feasible to integrate into undergraduate teaching. Embedding risk factor screening into curricula not only provides students with valuable clinical assessment skills but also fosters a preventative mindset that extends beyond the classroom (Dionisi et al. 2021). Using the example of fast bowlers, students can learn how to translate screening results into practical advice, exercise interventions and training modifications – skills that can later be applied to a range of populations, from athletes to workers to older adults at risk of falls.

The broader application of risk factor screening extends far beyond sports. Conditions such as low back pain, non-communicable diseases, musculoskeletal disorders in the workplace and age-related frailty all present with modifiable risk factors that can be identified early (Budreviciute et al. 2020; Klyne et al. 2022; Putsa et al. 2022; Wang, Hu & Wu 2022). Physiotherapy students trained in systematic screening are thus equipped not only to contribute to rehabilitation but also to support primary healthcare and public health initiatives. In South Africa, where resources for curative care are constrained, prevention has the potential to reduce the prevalence of avoidable disability and to alleviate strain on healthcare systems (Rural Health Advocacy Project n.d.).

Embedding prevention within undergraduate training aligns closely with the SDGs, particularly SDG 3 (good health and well-being), which emphasises health promotion, prevention of disease and improved population well-being (United Nations n.d.). By equipping students with preventative competencies, physiotherapy education can contribute directly to SDG 3 targets such as reducing premature mortality, strengthening primary healthcare and promoting lifelong health (Narain & Mathye 2019).

Training students to act as advocates for prevention at both individual and community levels enables physiotherapists to become agents of change in a healthcare landscape that increasingly prioritises sustainability and resilience (Misra et al. 2019; Sekome et al. 2023).

Taken together, these findings highlight the urgent need to reframe physiotherapy education in South Africa and globally. Prevention-focused teaching, informed by case studies such as the one presented in this article, has the potential to prepare graduates who are proactive rather than reactive and who can make tangible contributions to the health and well-being of populations. The integration of risk factor screening into undergraduate education represents not only a pedagogical innovation but also a professional responsibility to align physiotherapy training with the health priorities of the 21st century.

## Conclusion

This study identified modifiable risk factors for shoulder pain in amateur fast bowlers. These findings serve as an illustrative example of how undergraduate physiotherapy students could be taught to apply preventative screening principles in clinical practice. Strengthening preventative competencies in physiotherapy curricula may help reduce avoidable injuries and support SDG 3. By preparing graduates who are proactive in safeguarding health, physiotherapy education can contribute meaningfully to the reduction of preventable injuries and the promotion of well-being in diverse populations.
